# Differentiating Biomarker Features and Familial Characteristics of B-SNIP Psychosis Biotypes

**DOI:** 10.21203/rs.3.rs-3702638/v1

**Published:** 2024-01-05

**Authors:** David Parker, Rebekah Trotti, Jennifer McDowell, Sarah Keedy, Matcheri Keshavan, Godfrey Pearlson, Elliot Gershon, Elena Ivleva, Ling-Yu Huang, Kodiak Sauer, Scot Hill, John Sweeny, Carol Tamminga, Brett Clementz

**Affiliations:** Emory University School of Medicine; University of Georgia; Beth Israel Deaconess Medical Center; University of Texas Southwestern Medical Center; Rosalind Franklin University; University of Cincinnati; University of Texas southwestern medical center; University of Georgia

## Abstract

Idiopathic psychosis shows considerable biological heterogeneity across cases. B-SNIP used psychosis-relevant biomarkers to identity psychosis Biotypes, which will aid etiological and targeted treatment investigations. Psychosis probands from the B-SNIP consortium (n = 1907), their first-degree biological relatives (n = 705), and healthy participants (n = 895) completed a biomarker battery composed of cognition, saccades, and auditory EEG measurements. ERP quantifications were substantially modified from previous iterations of this approach. Multivariate integration reduced multiple biomarker outcomes to 11 “bio-factors”. Twenty-four different approaches indicated bio-factor data among probands were best distributed as three subgroups. Numerical taxonomy with k-means constructed psychosis Biotypes, and rand indices evaluated consistency of Biotype assignments. Psychosis subgroups, their non-psychotic first-degree relatives, and healthy individuals were compared across bio-factors. The three psychosis Biotypes differed significantly on all 11 bio-factors, especially prominent for general cognition, antisaccades, ERP magnitude, and intrinsic neural activity. Rand indices showed excellent consistency of clustering membership when samples included at least 1100 subjects. Canonical discriminant analysis described composite bio-factors that simplified group comparisons and captured neural dysregulation, neural vigor, and stimulus salience variates. Neural dysregulation captured Biotype-2, low neural vigor captured Biotype-1, and deviations of stimulus salience captured Biotype-3. First-degree relatives showed similar patterns as their Biotyped proband relatives on general cognition, antisaccades, ERP magnitudes, and intrinsic brain activity. Results extend previous efforts by the B-SNIP consortium to characterize biologically distinct psychosis Biotypes. They also show that at least 1100 observations are necessary to achieve consistent outcomes. First-degree relative data implicate specific bio-factor deviations to the subtype of their proband and may inform studies of genetic risk.

## Introduction

In psychosis, unique physiology and pathology aid diagnosis and promote targeting the most effective treatments to the needs of individual patients [1–3]. Currently, there is substantial neurobiological heterogeneity within and overlap between schizophrenia, schizoaffective disorder, and bipolar disorder with psychosis, the most prominent of the idiopathic psychoses. Since the inception of the of the Bipolar-Schizophrenia Network for Intermediate Phenotypes [B-SNIP; 4, 5, 6], we have aimed to improve the standard of care by identifying neurobiological features common across and unique to each of the prominent psychosis diagnoses.

To achieve these goals, we have used three characteristic approaches: (i) Collect sufficiently large “trans-diagnostic” samples across diagnoses to capture clinical and neurobiological heterogeneity; (ii) quantify biological and clinical features at multiple levels of analysis with an emphasis on capturing cognitive and physiological correlates of psychosis; and (iii) integrate over biomarkers that index a single construct (e.g., cognition, behavioral inhibition, auditory sensory registration) because no single measure adequately captures the underlying brain function. Even using large samples, multiple and multi-level biomarkers, and considering biomarkers both individually and as integrated statistical constructs, we found no obvious mapping of neurobiological features to schizophrenia, schizoaffective disorder, or bipolar disorder with psychosis at a clinically useful level [7, 8].

Following this result, B-SNIP modified the target to idiopathic psychosis generally. We modified our approach to probe whether there are similar subgroups within the larger trans-diagnostic psychosis sample. The first iteration of this program [B-SNIP1; 7] used numerical taxonomy with integrated behavioral, cognitive, and electroencephalography (EEG) biomarkers (called bio-factors) and identified three “psychosis Biotypes.” A second data collection and analysis effort (B-SNIP2; Clementz et al., 2022) re-quantified biomarker data from both B-SNIP1 and B-SNIP2 using updated and improved procedures. The outcome (i) replicated all steps in the biomarker and bio-factor quantification process, (ii) replicated psychosis Biotypes, (iii) cross-validated the subgrouping approach between B-SNIP1 and B-SNIP2, and (iv) construct validated Biotypes’ defining physiological features. These outcomes inspired testing whether B-SNIP-derived psychosis subgroups facilitate treatment targeting [9] and three major projects are currently ongoing. Using data from 3507 individuals, this paper aims to further improve and verify the practical clinical utility and robustness of this laboratory-centered approach to stratifying idiopathic psychosis cases.

First, a full B-SNIP-type laboratory evaluation is impractical for many settings. Previous psychosis Biotype algorithms combined information from multiple laboratory tests (e.g., multiple EEG paradigms estimated a single defining feature of Biotypes such as event-related potential [ERP] magnitude). To develop an efficient diagnostic procedure, however, each assessment must be separately evaluated to determine their individual utility and unique contributions. This paper accomplishes that goal.

Second, B-SNIP previously screened biomarkers for usefulness by applying statistical comparisons between DSM psychosis and healthy groups because there was no other generally accepted approach. That strategy missed informative biomarkers [e.g., 10]. In this paper, we remove that requirement, so the biomarker selection process is agnostic to clinical diagnoses. We also expanded the range of physiological assessments to include a direct measure of intrinsic neural activity [11], an important feature for differentiating psychosis cases [8, 12, 13].

Third, a drawback of numerical taxonomy is that it yields solutions regardless of whether subgroups are present and whether the outcomes are consistent. Previously, we estimated the number of subgroups using the gap statistic [14] and the preclustering step of SPSS’s TwoStep cluster analysis algorithm. In this paper, we expand to 24 such estimators of cluster number. In addition, for the best estimate number of clusters, we evaluate the consistency of assigning a subject to their modal group as a function of sample size used to derive the clustering solution. These analyses probe the robustness of the full sample solution and estimate the number of cases needed to construct a consistent biomarker-based diagnostic system.

Fourth, most psychosis cases in biomarker studies are medicated. Additionally, most have been chronically ill and chronically medicated. These factors create uncertainty whether biomarker differences between groups are related to trait illness, medications, or other effects of living with and adapting to a chronic condition. One way to address such concerns is to study the biological relatives without psychosis of the affected individuals. Medication and chronic condition effects have less explanatory power if the non-psychotic first-degree relatives show the same patterns of biomarker deviations as their ill relatives. In this paper, we show the responses across biomarkers in our large sample of first-degree relatives using our updated biomarker quantification procedures.

## METHODS

In the current B-SNIP database, there are 1907 psychosis cases, 705 nonpsychotic first-degree biological relatives of those cases, and 895 healthy persons recruited from the community. This is an increase of 479 psychosis and 423 healthy subjects over our previous numerical taxonomy paper [8], plus a re-analysis of biological relative data originally published (Clementz et al.[7]). The procedures for data collection and pre-processing are the same as Clementz et al. [8], but with updated and modified final quantification approaches.

B-SNIP recruitment sites were in Athens GA (University of Georgia and Augusta University Medical College of Georgia), Baltimore MD (Maryland Psychiatric Research Center), Boston MA (Beth Israel Deaconess Medical Center), Chicago IL (University of Illinois-Chicago and University of Chicago), Dallas TX (UT Southwestern Medical Center), Detroit MI (Wayne State University), and Hartford CT (Institute of Living). All recruitments, interviews, and laboratory data collections were completed at those locations. The Institutional Review Board at participating institutions approved the projects; participants provided informed consent prior to involvement.

Cases were drawn from academic and community mental health centers, small towns with large universities, large cities, inner cities, rural regions, affluent and less affluent areas. B-SNIP recruited a research sample, not an epidemiological sample; nonetheless, the large study numbers and broad geographical recruitment foster generalizability of the outcomes across the range of early onset through midcourse to chronic idiopathic psychosis. See Table 1 for demographic information of probands and healthy comparison subjects. See table S3 for demographic information of first-degree relatives.

### Clinical Evaluations

B-SNIP recruitment details and approaches are available in Tamminga et al. [6]. Briefly, clinically stable outpatients were administered the Structured Clinical Interview for DSM diagnosis [DSM-IV-TR; 15]. Psychosis cases were limited to schizophrenia (n = 783), schizoaffective disorder (n = 582), and bipolar I disorder with psychosis (n = 542) because these are the diagnoses with the highest prevalence in most settings. Cases were rated on the Birchwood Social Functioning [SFS; 16], Montgomery-Asberg Depression Rating [MADRS; 17], Positive and Negative Syndrome [PANSS; 18, 19], and Young Mania Rating [YMRS; 20] scales. Healthy persons were free of lifetime psychosis syndromes, recurrent mood syndromes, and a history of psychosis or bipolar disorders in their first-degree relatives. Table S1 shows the clinical information by group. Table S2 provides a summary of medication information. We previously demonstrated that medication effects do not significantly account for group differences on biomarker features [see 8].

### Biomarker Panel

Participants completed comprehensive laboratory evaluations within a few weeks of their clinical assessments. Papers on the individual laboratory paradigms provide extensive data collection and analysis details [10, 21–29]. Details of biomarker quantification and numerical taxonomy procedures are in Clementz et al. [8].

The laboratory measures used for Biotypes creation are traditional endophenotypes [30]. Each paradigm has a substantial literature supporting its use as a biomarker of psychosis. Paradigms include (i) the Brief Assessment of Cognition in Schizophrenia [BACS; 31, 32] to test general cognitive performance, (ii) pro- and anti-saccades [saccades; 33, 34, 35] to assess speed of visual orienting, goal maintenance, and inhibitory control under perceptual conflict, and (iii) a stop signal task [SST; 36] to assess adequacy of adapting speeded motor responses to situations requiring inhibitory control [22, 23].

There are also three assessments of brain physiology as measured with dense-array electroencephalography (EEG). Event-related brain potentials (ERP) were measured with (iv) auditory paired stimuli and (v) auditory oddball paradigms [37–40] to assess preparation for and recovery from sensory activations, neural responses to stimulus salience and relevance, context updating in working memory, and nonspecific (or intrinsic) brain activity during performance (i.e., brain activity not time-locked to stimulus processing). The 9–10 second inter-pair interval of the paired stimuli paradigm was also included as a direct measure of (vi) intrinsic EEG activity, or IEA [i.e., background brain activity not associated with ongoing stimulus processing requirements; 10].

### Data Reduction and Creation of Bio-Factors

Within each laboratory measurement domain (BACS, saccades, SST, paired stimuli ERP, oddball ERP, IEA/EEG), principal component analysis (PCA; Covariance Matrix, Promax Rotation, Kaiser Normalization, Kappa = 3) reduced multiple variables within that domain to an efficient and smaller variable set. This was done for two main reasons. First, for estimating the true value on any theoretical construct, multiple independent measures are better than any single variable. For example, neural response to stimulus salience is better estimated by many ERP measures than by a single voltage from a single sensor at one time point. Second, reducing the redundancy of measurements increases the accuracy of numerical taxonomy [41].

All psychosis and healthy participants were included in PCAs using standardized variables. Age and sex-adjusted biomarker data were used, if such effects were statistically significant, based on the procedure described in Dukart et al. [42], and previously described in Clementz et al. [8]. This approach produced variables integrated over multiple biomarker measurements, which were the units of analysis for numerical taxonomy. We call these PCA variates “bio-factors” since they capture multiple facets of neuro-cognition and physiological responses and labelled them based on their most characteristic biomarker associations. Similar subcomponents of these procedures proved to be stable and replicated with high accuracy in two independent samples, each of which contained > 700 psychosis and > 200 healthy participants [8]. Nonpsychotic first-degree relatives’ bio-factor scores were created by applying the PCA coefficients obtained from psychosis and healthy persons.

#### BACS.

The BACS subtests, covering verbal abilities, processing speed, reasoning, problem solving, and working memory, were scored according to standard procedures. PCA of the BACS subtests identified one component. Thus, there is one BACS bio-factor.

#### Saccades.

Participants completed three pro-saccade (gap, synchronous, and overlap) and one overlap anti-saccade condition [26, 29, 34, 43]. Trials were scored for (i) direction (to evaluate correct or error response) and (ii) onset latency. Pro-saccade latencies, anti-saccade latencies, and proportion of correct anti-saccades were included in the PCA. The scree identified two bio-factors called “latency” and “antisaccade.”

#### Stop Signal Task.

A baseline task of go-only trials, with a visual stimulus presented pseudo-randomly to the left or right of central fixation, assessed baseline reaction time. For stop-signal trials, a go cue appeared to the left or right. On 40% of trials, a stop signal was presented at central fixation [22, 23].

Participants were instructed to respond quickly and accurately to the go cue unless they encountered the stop signal. Strategic slowing (difference between response latencies on baseline go trials and go trials during stop signal performance) and proportion of stop signal errors were included in the PCA. The scree identified one SST bio-factor.

#### Auditory ERP tasks.

For the paired-stimuli task, participants passively listened through headphones to at least 120 broadband auditory click pairs with 500 msec inter-click interval occurring every 9.5 sec on average (9–10 sec inter-pair interval). For the oddball task, participants listened through headphones to 567 standard (1000 Hz) and 100 target (1500 Hz) tones presented in pseudorandom order (1300 msec inter-trial interval) and pressed a button when a target was detected (to maintain vigilance).

Data from trials free of artifacts (± 75 mV) were averaged to create 64-sensor ERPs. In addition to analyzing the grand-averaged ERP in the time domain, a frequency-wise PCA of evoked power [21, 24] empirically defined low, beta, and gamma frequency bands. The combination of temporal and frequency information over all sensors and time points maximizes the use of spatial, temporal, and oscillatory information. A spatial PCA [21, 24, 44, 45] on the grand-averaged ERP and each frequency band yielded four waveforms (“virtual sensors”). These virtual sensors were analyzed instead of separate sensors, efficiently summarizing the spatial distributions, minimizing the number of statistical comparisons, and maximizing the signal/noise ratio of the EEG/ERP data [44] (See figure S1 for EEG response across time for each stimulus type). For the paired-stimuli task, the PCA scree identified three paired-stimuli bio-factors (figure S2). For the oddball task, the PCA scree identified three oddball bio-factors (figure S3).

#### Intrinsic EEG Activity (IEA).

Data derived from the 9–10 sec inter-pair interval of the paired-stimuli task [10]. No stimuli were presented during this period. EEG data were pre-processed following methods described above and in Thomas et al [10]. Data were transformed into the frequency domain, with frequency bands empirically determined using PCA [10], resulting in four primary bands: delta/theta, alpha, beta, and gamma. The PCA scree identified one IEA bio-factor.

### Data Analyses

#### Clustering:

The 11 bio-factors were used to construct psychosis subgroups via k-means clustering [8]. Only psychosis cases were used at this stage since the goal was to determine how to meaningfully parse bio-factor variance within psychosis. The number of clusters given the data were determined by the gap statistic [14] and the 23 estimators in the NBclust package in R [46].

#### Clustering Membership Consistency

Bootstrapped samples of psychosis cases were selected at sizes of 500 to 1800 cases, in 100 case increments, with 1000 pseudo-replicates for each sample size. Each of the clustering solutions were then compared to the raw total sample solution using unadjusted and adjusted rand indices for the least and most conservative estimates of cluster membership consistency.

#### Group Differences:

The 11 bio-factors were compared between-groups using analysis of variance, with Tukey’s method for post-hoc evaluations (HSD or Tukey-Kramer where appropriate). For statistical significance in omnibus tests, the Holm-Bonferroni procedure [47] was used to maintain the family-wise alpha at .05. For first-degree relatives’ comparisons, degrees of freedom were adjusted based on the number of unique families included in the analysis since some families in the study had multiple members.

## RESULTS

### Bio-factors by Psychosis and Healthy Groups

The means, standard deviations, and effect sizes for the total psychosis versus healthy groups are presented in Table 2. Of the 11 bio-factors, those in the cognition set (BACS, antisaccade, SST) differentiated the best (F’s > 90.7, p’s < .001, Glass Δ’s of −1.04, −1.03, and − .45). The ERP response magnitude bio-factors also significantly differentiated groups (F’s > 33.6, p’s < .001), but with considerably less separation (Glass Δ’s of −0.35 and − 0.25); the same was true of the intrinsic activity bio-factors (F’s > 12.3, p’s < .001, Glass Δ’s of 0.19, 0.26, and 0.16). The bio-factors in the stimulus salience set were more modestly differentiating, with the latency bio-factor failing to separate psychosis and healthy groups (F < 1, p > .770, Glass Δ = − .01). The other two stimulus salience bio-factors, P300 complex and paired-stimuli S2, showed significant effects (F’s of 6.2 and 13.8, p’s of .013 and < .001) of small group differentiations (Glass Δ’s of 0.09 and − 0.16).

### Number of Clusters

These analyses addressed the best estimate of the number of clusters given individual participant scores across the 11 bio-factors. Gap statistic figures are presented in figure S4, and results from the 23 cluster number estimators of the NBclust packag are presented in table S4. Both the Gap statistic and NBclust majority rule indicate the most parsimonious solution is three clusters. Therefore, k-means was obtained requesting a three-cluster solution; the algorithm achieved cluster stability within 43 iterations [see 8]. The k-means outcome resulted in ~ 630 observations per cluster (psychosis Biotypes) as described in Table 1 (BT1 n = 630, BT2 n = 631, BT3 n = 646).

### Consistency of Cluster Membership Assignment

**Figure 1:**
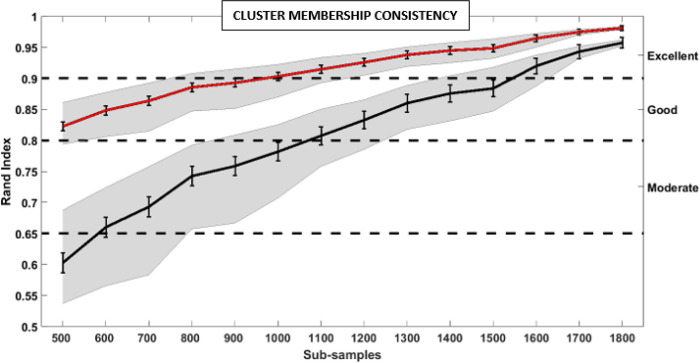
Cluster Consistency To test cluster member consistency, a bootstrapping approach was used on subsamples in sizes ranging from 500 to 1800, in steps of 100, of the total sample (1000 iterations each step). A random subsample was selected at each sample number, normalized, and submitted to the k-means clustering algorithm 1000 times. Membership from each subsample clustering solution was compared to the membership of the total sample with an adjusted and non-adjusted rand index. Rand index is a similarity measure between two clustering solutions. Red line is the average of the non-adjusted rand index the 1000 iterations at each subsample. Error bars are the 99% Confidence interval. Gray shading represent the 40^th^ and 60^th^ percentile values. Black line is the adjusted rand index average across the 1000 iterations at each sub-sample. At sample sizes greater 1000, the adjusted rand index shows good and the unadjusted rand index shows excellent consistency across subsamples.

Figure 1 shows two consistency estimates for k-means membership using a sub-sampling approach (1000 iterations at each subsample size from 500 to 1800 probands). The first rand index (upper red line) shows consistency with the full model solution without adjusting for chance assignment. This outcome shows remarkable consistency of > 95% agreement for samples of greater than 1400 observations, excellent agreement of > 90% for sample sizes of greater than 900, and still good agreement > 82% for sample sizes of at least 500. The second rand index (lower black line) shows consistency adjusting for the probability of a case being assigned by chance to one of the three groups. This most conservative metric shows remarkable agreement of > 95% for samples of greater than 1600, excellent agreement of > 90% for samples of greater than 1500, and good agreement of > 80% for samples of greater than 1000.

### Bio-Factors by B-SNIP Psychosis Biotypes

**Figure 2:**
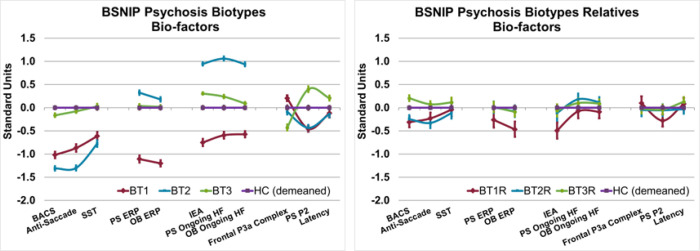
Bio-factors by B-SNIP Psychosis Biotypes and their Relatives **Right:** Standardized values of each of the 11 bio-factors that were included in the clustering algorithm by Biotype. The average healthy values was subtracted from each bio-factor to show the relative difference across measures. Across all bio-factors there were significant differences betweene psychosis Biotypes (Holm-Bonferroni adjusted significance, F’s = 20.21 to 703.81, p’s < .001). Error bars=99% Confidence intervals. **Left:** Standardized values of each of the 11 bio-factors by non-psychotic first degree relatives separated by their proband Biotype membership. BACS, antisaccade, paired stimuli and oddball ERPs, and IEA bio-actors differentiate the relative groups (Holm-Bonferroni adjusted significance, F’s > 4.1, p’s <.007). Error bars=99% Confidence intervals.

Figure 2 (left plot) shows bio-factors plotted by group membership. All bio-factors differentiate psychosis Biotypes (Holm-Bonferroni adjusted significance, F’s = 20.21 to 703.81, p’s < .001). This result is not surprising because numerical taxonomy used these bio-factors to create maximally homogeneous and distinct groups. When adding the healthy group to the models, all comparisons remained significant and of similarly large magnitude (Holm-Bonferroni adjusted significance, F’s = 14.96 to 442.78, p’s < .001). The differences in bio-factor pattern for psychosis groups in comparison to the healthy group is highly consistent, but more robust than previous reports [8]. Consequently, we maintained the same designations of BT1 (low cognition and low neural response magnitudes), BT2 (low cognition, poor inhibition, accentuated intrinsic brain activity), and BT3 (reasonably normal across most bio-factors but mildly deviant on measures of stimulus salience).

**Figure 3:**
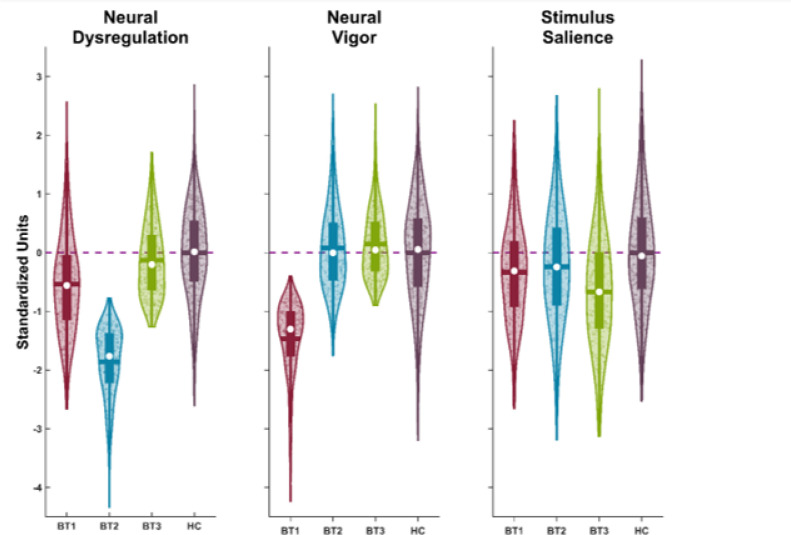
Canonical Discriminant Analysis Variates by B-SNIP Psychosis Biotypes Results of the canonical discriminant analysis which had 3 significant variates (chi-squares > 110.7, p’s<.001; canonical correlations of .69, .64, and .26, p’s <.001). **A.** CDA variate 1 (Neural dysregulation) was associated with lower cognitive scores and higher ongoing frequency activity from the Oddball and Paired Stimulus tasks. **B**. CDA Variate 2 (Neural Vigor) was associated with reduced ERP responses and intrinsic neural activity. **C.** CDA variate 3 (Stimulus Salience) was associated with the frontal P3a EEG response, Paired Stimulus S2 ERP activity, and pro-saccade Latency. See table S5 for CDA Structure Matrix.

Biotypes have unique patterns across the 11 bio-factors. They are also distinguished from healthy persons on these variables. To ease visualization of unique group bio-factor patterns, we used canonical discriminant analysis (CDA) with the criterion of group membership (BT1, BT2, BT3, healthy) and the 11 bio-factors as predictors. This is a simplification of group differentiations in the 11-variable space of the bio-factors. The CDA yielded three significant variates (chi-squares > 110.7, p’s < .001; canonical correlations of .69, .64, and .26, p’s < .001; see Fig. 3 and table S5).

CDA Variate 1, what we termed “neural dysregulation,” has the most significant associations with antisaccades (r = .60), BACS (r = .56), and ongoing EEG high frequency activities (oddball r = .48, paired-stimuli r = .47). Lower scores indicate a trio of poor cognitive performance and behavioral inhibition combined with accentuated background brain activity during stimulus processing. Neural dysregulation best distinguishes BT2 from the other groups, with post-hoc tests showing a pattern of BT2 < BT1 < (BT3 = HC). CDA Variate 2, termed “neural vigor,” best separates BT1 from the other groups and is associated with a reduced ERP responses (paired-stimuli r = .69, oddball r = .72) and reduced intrinsic EEG activity (IEA r = .70). Lower scores indicate generally reduced neural activity. Post-hoc comparisons show a pattern of BT1 < (HC = BT2) < (BT2 = BT3). CDA Variate 3, termed “stimulus salience,” best separates BT3 from the other groups and is associated with frontal P3 complex responses (r = .61), response to the second stimulus of the paired-stimuli paradigm (r = − .57), and prosaccade latencies (r = − .34). Lower scores indicate altered sensitivity to stimulus salience in comparison to healthy persons. Post-hoc comparisons show a pattern of BT3 < (BT2 = BT1) < H.

### First-Degree Relatives by Proband Cluster Membership

Figure 2 (right plot) shows bio-factors plotted for the non-psychotic first-degree relatives by the proband to whom they are related. The BACS, antisaccade, paired stimuli and oddball ERPs, and IEA bio-factors differentiate the relative groups (Holm-Bonferroni adjusted significance, F’s > 4.1, p’s < .007). The relatives’ patterns of deviation on those bio-factors are like the patterns among their probands. The BACS [(BT1 = BT2) < H < BT3; effect sizes for relatives versus healthy of BT1 = −0.33, BT2 = −0.28, and BT3 = 0.26] and antisaccade bio-factors [(BT1 = BT2) < (H = BT3); effects sizes of BT1 = −0.30; BT2 = −0.44, and BT3 = 0.15] show similar patterns of deviations, except that BT3 relatives had the best general cognitive performance, even in comparison to healthy persons. The patterns for the ERP magnitude measures recapitulate the probands’ patterns with BT1 relatives having lower ERP amplitudes than the other three groups combined (paired stimuli ERP effect sizes of BT1 = −0.29; BT2 = 0.01, and BT3 = 0.01; oddball ERP effect sizes of BT1 = −0.47; BT2 = −0.08, and BT3 = −0.08). Likewise, the IEA bio-factor shows the same pattern as the ERP magnitude measures, with BT1 relatives being lower than the other three groups (effect sizes of BT1 = −0.63; BT2 = −0.16, and BT3 = −0.08).

## DISCUSSION

The B-SNIP consortium reported and replicated [8] that DSM schizophrenia, schizoaffective disorder, and bipolar disorder with psychosis are not obviously neurobiologically distinctive, a conclusion also supported by large-scale genetics projects [48]. Such realities muddy efforts to link a specific clinical feature or syndrome to identify a pathology responsive to a specific intervention. Guze [1] believed standardized clinical evaluations in psychiatry are useful for detecting specific disease entities, but only “up to a point.” As the “all psychosis” versus “healthy” comparisons reveal, much is hidden by imprecise groupings. For psychosis, including neuroscience in diagnostic definitions could be beneficial since the brain is the most affected organ. B-SNIP implemented laboratory assessments to stratify idiopathic psychosis cases regardless of their specific clinical diagnoses [7, 8]. We demonstrated that these psychosis Biotypes have unique patterns of clinical features that can assist laboratory diagnosis [49].

This paper modifies and extends our previous efforts. We improved the efficiency and reinforced the robustness of the B-SNIP psychosis Biotypes algorithm and identification in three ways:

1. Previous iterations required multiple laboratory paradigms to assess a single construct (e.g., the n100 response in an auditory ERP). We changed to using paradigms individually. This approach (i) provides internal replication of effects common to multiple paradigms, and (ii) aides identification of efficient tests for distinguishing psychosis subgroups. We confirm that auditory paired-stimuli and oddball ERPs have the same patterns across psychosis Biotypes. Three measures of background or intrinsic brain activity (IEA, and ongoing activity during the paired-stimuli and oddball tasks) also showed the same Biotypes differentiations. A significant project that we have worked on is to develop the “ADEPT” algorithm which aims to evaluate the diagnostic utility of these bio-factors individually [49]. This could help prioritize the order measures should be collected and would help with future clinical implementation. These similar laboratory measures, may track differently across interventions, thus providing unique treatment targets and outcome assessments.
2. In previous iterations, biomarker selection was tied to statistical differences between healthy and DSM diagnostic groups. Here, biomarker inclusion is agnostic to group membership and is instead based on characteristics of the measures themselves (e.g., the prototypical patterns of EEG/ERP responses to auditory oddball stimuli). We expanded the physiological assessments to include a direct measure of intrinsic neural activity (IEA). This approach yielded two additional bio-factors (IEA and separate measures of ongoing activity from the paired stimuli and oddball paradigms), rearranged what is measured by individual bio-factors (i.e., paired-stimuli and oddball ERPs disentangled, frontal P3 ERP uniquely quantified), and enhanced separations between psychosis Biotypes. We also extracted three components from the 11 bio-factors that uniquely identified the most characteristics features of psychosis Biotypes from each other and from healthy comparison subjects: neural dysregulation for BT2, neural vigor for BT1, and deviations of stimulus salience for BT3. The discrimination of BT3 was a new addition made possible by this modified approach.
3. For procedures like k-means, it is crucial to validate the veridicality of the number of subgroups. In comparison to previous psychosis Biotypes iterations, we used one overlapping (gap statistic) and 23 new cluster estimation procedures (from NBclust in R). The most probable outcome was again three subgroups (10 of 24 estimates). We also evaluated the consistency of assigning cases to their modal subgroup across a range of bootstrapped sample sizes. The bio-factors are highly repeatable [8], so the chance group assignment correction of the adjusted rand index is conservative for our case. Nevertheless, even using a conservative approach, individual cases are assigned to their modal Biotype group with exceptional to good consistency with sample sizes greater than 1000. The outcome of this analysis, however, highlights the sample sizes required to construct a robust neuroscience-assisted classification procedure.

The analysis of biological relatives’ bio-factors probed which neuro-biological features may be crucial to the etiology and pathogenesis of B-SNIP psychosis Biotypes. Bio-factor patterns of probands were replicated among their nonpsychotic first-degree relatives for BACS, antisaccade, auditory ERPs and IEA bio-factors. Given that these variables also show high familial similarity [more liberally called heritability; 7], these measures appear to be endophenotypes [30], but only for specific psychosis Biotypes. Even BT3 psychosis cases, who are statistically equivalent to healthy persons on cognitive performance, are deficient in relation to their non-psychotic family members. This finding may refine previous reports of cognitive endophenotypes for bipolar disorder [50, 51], and also recapitulates the generally lower cognitive performance of psychosis probands in relation to their unaffected first-degree relatives [52]. These outcomes raise the possibility that laboratory measures such as motor inhibition from the SST, induced EEG activity, and stimulus salience assessments maybe pathology markers of idiopathic psychosis after it has developed rather than causal markers related to elevated psychosis-risk. Alternatively, the nonfamilial bio-factors may index other acquired deviations that explain why members of the same high-risk family differ in psychosis manifestation.

B-SNIP’s multi-domain, neurobiologically defined, trans-diagnostic psychosis Biotypes offer an alternative to the traditional clinical approach for case stratification. This approach has been galvanized by the extent of unsuccessful research into the biological mechanisms of conventional psychosis diagnoses and the failure to develop new treatments. With our refined biologically based sub-groups, we have attempted to capture homogenous disease groups with common biological dysfunctions. This is an approach we will continue to refine and is being more widely adopted in biological psychiatry research [53–56]. Critically, it deserves thorough and rigorous testing. The bio-factor underpinnings of B-SNIP Biotypes are stable and replicable, and the Biotypes themselves show excellent cross- and construct validation [8, 12, 13, 57–59]. They also have differentiating patterns of clinical features [9, 49, 60].

This does not mean B-SNIP psychosis Biotypes are the final or correct model; the solution is a function of variables used and methods applied. But this approach yields rational, testable hypotheses [61, 62] of pathophysiological theories and treatment targets that are not derivable from any available alternative. It perhaps offers a beginning for transitioning a part of psychiatry to a laboratory discipline. The ability to tailor treatments to individual psychosis patients and improve outcomes, however, will be the ultimate validator of this or any other approach to psychosis diagnosis [63].

## Figures and Tables

**Table 1:** Demographic Characteristics of Biotypes

Characteristic	Biotype-1 n = 630	Biotype-2 n = 631	Biotype-3 n = 646	Healthy n = 895
**Mean Age (SD)**	38 (13)	39 (12)	36 (12)	35 (12)
**Sex**				
Male	57%	44%	51%	43%
Female	43%	56%	49%	57%
**Ethnicity**				
Not Hispanic	86%	88%	90%	88%
Hispanic	14%	12%	10%	12%
**Race**				
Black	50%	42%	24%	29%
American Indian	0.8%	0.2%	0.3%	0.2%
Asian	3.4%	1.9%	3.6%	8.3%
White	39%	47%	64%	56%
Multiracial	4.6%	5.6%	5.0%	3.6%
Hawaiian/Pacific Islander	0%	0%	0%	0.2%
Other	2.6%	2.9%	3.0%	2.6%
**Global Functioning (SD)**	52 (12)	51 (12)	56 (14)	85 (7)
**Mean Proband SES (SD)**	50 (14)	49 (14)	43 (15)	35 (14)
**Mean Family SES (SD)**	44 (16)	44 (16)	38 (16)	37 (15)

**Table 2:** Standard Score Mean (SD) of Bio-factors by Psychosis and Healthy

Bio-Factors	psychosis	healthy	test Result	GLASS DELTA Effect size
BACS	−0.25 (0.97)	0.58 (0.80)	P < H	−1.03
Anti-saccade	−0.23 (1.02)	0.51 (0.73)	P < H	−1.02
SST	−0.15 (0.99)	0.28 (0.96)	P < H	−0.45
PS ERPs	−0.08 (1.01)	0.16 (0.95)	P < H	−0.26
OB erps	−0.11 (0.98)	0.23 (0.98)	P < H	−0.35
intrinsic EEG	0.06 (1.03)	−0.10 (0.84)	P > H	0.19
pS ongoing hf	0.08 (1.03)	−0.16 (0.91)	P > H	0.26
ob ongoing hf	0.05 (0.97)	−0.09 (0.88)	P > H	0.16
frontal P3 complex	−0.03 (0.97)	0.07 (1.04)	P < H	−0.10
PS S2	−0.05 (1.00)	0.11 (0.97)	P < H	−0.16
Latency	0.00 (1.06)	0.01 (0.86)	P = H	−0.02
